# Cardiovascular Morbidity in Ankylosing Spondylitis: A Focus on Inflammatory Cardiac Disease

**DOI:** 10.7759/cureus.25633

**Published:** 2022-06-03

**Authors:** Pradnya Brijmohan Bhattad, Mugdha Kulkarni, Parasbhai D Patel, Mazen Roumia

**Affiliations:** 1 Cardiovascular Medicine, Saint Vincent Hospital, UMass Chan Medical School, Worcester, USA; 2 Internal Medicine, BronxCare Hospital, Bronx, USA; 3 Internal Medicine, East Tennessee State University, Quillen College of Medicine, Johnson City, USA

**Keywords:** inflammatory cardiac disease, cardiovascular mortality, seronegative spondyloarthropathy, spondyloarthritis, ankylosing spondylitis

## Abstract

Ankylosing spondylitis (AS) is associated with an increase in cardiovascular (CV) morbidity when compared to the general population. The increased risk of CV involvement in AS is likely multifactorial including inflammation accelerating atherosclerosis and the cardiac inflammation itself in the form of aortitis and conduction anomalies. Establishing indisputable evidence linking AS and CV disease is challenging due to AS being relatively rare and it affects 1:1,000 and all studies analyzing the association between AS and CV disease involve a small sample size making long-term outcome measurements limited. The article reviews the literature studying the association between AS and CV disease as well as the impact of therapies for AS on the CV system (CVS).

## Introduction and background

Ankylosing spondylitis (AS) is a subset of the spondyloarthropathies, which include psoriatic arthritis, reactive arthritis, spondyloarthritis associated with inflammatory bowel disease, and undifferentiated spondyloarthritis [1,2]. The main site of inflammation in AS is the axial skeleton, and patients usually have characteristic lower back pain and associated low back stiffness, which can severely affect a patient’s functionality and quality of life [3]. The common therapies for AS include nonsteroidal anti-inflammatory medications (NSAIDs) and physical therapy, with tumor necrosis factor inhibitors (TNF inhibitors) also playing a role as a first-line in some cases, were previously used in refractory cases but not enough evidence currently for use in refractory cases [3-5]. AS shows a striking correlation with HLA-B27. HLA-B27, a class I surface antigen encoded by the B focus on the major histocompatibility complex (MHC) has a prevalence of 75%-90% in patients with AS [2-4]

The age of onset of AS seems to correlate with whether the patient is HLA-B27 positive or negative, HLA-B27 positive patients become symptomatic earlier at an average age of 24.8 years, while HLA-B27 negative patients become symptomatic at an average age of 27.7 years [5]. AS is more common in males than females in an approximately 2:1 ratio [3]. Prevalence was found to range from 0.1% to 2% in a study conducted by Gran and Husby [6]. As a rule of thumb, AS occurs in 0.2% general population, 2% of HLA-B27 positive individuals, and 20% of HLA-B27 positive people with a family history of AS [7]. Clinical features of AS include inflammatory back pain, peripheral joint arthritis predominantly in the lower extremities, anterior uveitis, and enthesitis [7,8]. According to the Assessment of SpondyloArthritis International Society (ASAS) criteria, “inflammatory back pain” typically exhibits at least four of the following five features: a) age of onset <40 years, b) Insidious onset, c) improvement with exercise, d) no improvement with rest, and e) pain at night (with improvement upon arising). Juvenile-onset AS is associated with more peripheral joint manifestations, while adult-onset AS is associated with axial joint manifestations [8]. Patients often lose spinal mobility due to inflammation and stiffness and can develop hyperkyphosis [8]. Long-term sequelae can include osteoporosis and an increased risk of bone fractures [2,8]. Cardiac involvement in AS may cause symptoms such as dyspnea, and fatigue and can be wrongly attributed to the patient’s rheumatic disease [9].

## Review

Diagnosis of AS

Inflammatory back pain occurs in 70%-80% of patients with AS and was one of the three criteria for diagnosis of AS listed in the modified New York criteria [10,11]. Even though these criteria are specific, they are insensitive for diagnosing early disease [12]. In 2009, the ASAS formulated classification criteria for axial spondyloarthritis (SpA) that was based on imaging, clinical, and laboratory criteria [13].

Pathophysiology

The pathogenesis of AS is thought to be immune-mediated, supported by evidence of an association with HLA-B27 [7,14]. This theory is based on an autoimmune-like picture of AS, where T-cells and macrophages are found in joint biopsies of patients with AS [15]. AS is associated with higher expression of T-bet, a transcription factor [16]. Several cytokines including TNF-α, IL-17, and IL-23 play an important role in the disease process with affected sacroiliac joint being infiltrated with CD4+, CD8+ cells, and macrophages [2,16]. An elevated acute phase response may be present, including an elevated erythrocyte sedimentation rate (ESR) and elevated C-reactive protein (CRP), in approximately 50% to 70% of patients with active AS, but such changes are less frequent in patients with nonradiographic axSpA (nr-axSpA) at approximately 30% [17]. The core pathology in AS is still believed to be an interaction between bacteria and HLA-B 27 [18,7]. HLA-B27 could play a role by presenting an unknown arthritogenic molecule, causing subsequent inflammation in certain joints of the body [18].

AS and cardiovascular (CV) disease

AS has increased CV morbidity and mortality as observed by many researchers [3,4,19]. Despite multiple studies on CVS mortality in AS, it is unclear if the increased mortality is a result of cardiac manifestations of AS itself or a concomitant increase in CV diseases secondary to accelerated atherosclerosis in AS. The standardized mortality rate (SMR) was significantly elevated for hypertensive diseases and diabetes mellitus (DM) in AS [20]. In AS, vascular morphologic and functional abnormalities are seen along with inflammation-associated endothelial injury, collectively leading to atherosclerosis [21]. In a study published in 2005 on 27 patients with AS compared with 19 control subjects, even after correcting for risk factors such as smoking history and elevated body mass index (BMI), patients with AS had elevated levels of the proinflammatory cytokine IL-6 (ninefold increase) and CRP ( sixfold increase), which are systemic inflammatory markers [22]. The inflammatory process promotes endothelial injury, which in turn leads to formation of atheromas and continued development of atherosclerosis [21]. IL-6 induces the acute phase response, leading to elevated CRP, and fibrinogen along with activation of monocytes. The activated monocytes deposit fibrinogen in the blood vessel wall, contributing to the formation of an atheroma. Furthermore, IL-6 and other inflammatory cytokines are released by foam cells and smooth muscle cells when the endothelium is damaged, leading to even more blood vessel damage. IL-6 interacts with the hypothalamus-pituitary-adrenal (HPA) axis, influencing cardiac risk factors including insulin sensitivity, elevated BMI, and elevated blood pressure [2]. According to a review article published in 2010, patients with AS tended to have lower total cholesterol and lower high-density lipoprotein (HDL) levels [19]. There is an inverse relationship between IL-6 levels and cholesterol and HDL levels [22]. As per Khovidhunkit, low density lipoprotein (LDL) cholesterol levels decrease during inflammation but there is an appearance of small dense LDL which is thought to be more proatherogenic [23,24]. The connection between inflammatory cytokines and accelerated atherosclerosis has been discussed in an article published in 2003.The cytokines circulating throughout the body affect distant tissues (adipose tissue, skeletal muscle, liver, vascular endothelium), leading to a myriad of downstream consequences, which include events such as increased insulin resistance, dyslipidemia, increase in prothrombotic state , oxidative stress, and endothelial dysfunction [25]. When looking at AS in particular, patients with milder disease courses (as determined by the Bath AS Disease Activity Index) had measurements of carotid and femoral artery plaque deposition that did not appear to support evidence of accelerated atherosclerosis [26]. However, in a meta-analysis performed involving 521 patients with AS compared with 445 matched controls, patients with moderate to severe AS, were found to have a significant increase in carotid intima-media thickness, which is an indicator of accelerated atherosclerosis [26]. Even when controlled for traditional CV risk factors, AS shows increased risk of atherosclerosis [27]. In patients without clinically evident CV disease, carotid intima-media thickness could predict macrovascular disease and appeared to correlate with ESR levels and duration of disease. In light of the link of inflammation in AS and worsening CV mortality, the use of anti-inflammatory agents is a subject of considerable interest for both treating AS and mitigating the development of CV sequelae [28]. In a study comparing the prevalence of CV diseases and risk factors in RA, psoriatic arthritis (PsA) and AS, the prevalence ratio of ischemic heart disease (IHD) (1.2), peripheral vascular disease (1.6), congestive heart failure (1.8), cerebrovascular disease (1.7), type 2 DM (1.2), hyperlipidemia (1.2), and hypertension (1.3) were higher in patients with AS compared to controls [29]. This helps understand the etiology behind increased CV disease prevalence in AS patients. One large Canadian population-based study of 21,473 patients with AS found that patients with AS are at an increased risk of vascular disease (43% higher), cerebrovascular disease (60% higher), and increased CV mortality (35% higher). Independent predictors of vascular mortality include age, gender, income, dementia, and peripheral vascular disease (PVD). Males have approximately double the risk of females, and those with PVD have a sixfold increase in mortality [3]. The reason why the mortality rate in males with AS is much higher than that in females is not well understood. A possible theory is that the disease is milder with less inflammation in females, as supported by lower CRP levels in females than males [30]. Another theory involves the protective estrogen effect in premenopausal women, since AS manifests at younger ages [3]. Many reasons could explain the higher risk of vascular mortality in AS. Patients with chronic inflammatory conditions are at a higher risk for metabolic syndrome. Metabolic syndrome causes biochemical changes via inflammatory cytokines, which could contribute to the onset and persistence of AS and other inflammatory arthropathies [31]. The medical management of AS including NSAIDS, DMARDS (sulfasalazine, TNF-α inhibitors) and biologics like IL-17 inhibitors influence CV manifestations as well. NSAIDS adversely impact CV outcomes in AS and trials of IL-17 inhibitors show CV morbidity in some patients [1,32].

Inflammatory cardiac disease associated with AS

There are three types of inflammatory cardiac diseases that are associated with AS, which include aortic insufficiency and aortitis, conduction abnormalities, and myocardial involvement affecting left ventricular ejection fraction. In a review of the cardiac manifestations of AS, histopathological features of aortitis were found to be similar to that found in syphilis [33]. In a retrospective study performed on 40 patients with AS, aortic insufficiency was found in approximately 12.5%, atrioventricular block in 7.5%, and a bundle branch block 12.5% of patients with AS [34]. Subaortic fibrous ridging was found even before signs of aortic regurgitation [35]. However, as observed in a small study, AS is not associated with an increased risk for structural heart disease [36].

AS, myocardial ischemia, and infarction

Although elevated CV risk has been proven to exist in patients with chronic inflammatory rheumatic arthritis, studies on AS and IHD have been contradictory. There have been multiple studies that show an elevated risk for IHD and acute myocardial infarction (MI) in patients with AS, but several studies have been unable to reproduce these original results, suggesting poor study design or population selection [37].

In a meta-analysis published in 2011, the number of MIs in AS patients over a follow up period averaging 22 years was examined. The incidence of MI in AS patients was 7.4%, while the incidence in the control group was 4.6%, but this did not achieve a level of significance in the meta-analysis [38,30]. A retrospective cohort study involving 1,686 patients with AS found a hazard ratio of 1.28 for MI, but once again, it did not achieve a level of significance [39]. Another study published in 2013 involving 4,794 patients with AS found that the crude and adjusted hazard ratio for the development of IHD was 1.47 with an average follow up time of 31.9 months [40].

CV disease is thought to be more prevalent in patients with AS due to the systemic inflammatory nature of AS, decreased levels of HDL cholesterol, increased prevalence of metabolic syndrome and the long-term use of NSAIDs in the treatment of AS. The chronic use of NSAIDs may contribute more to the development of IHD in patients with AS than the effects of AS itself [37,40]. Although there appears to be an increased age-adjusted risk in females with AS for the development of IHD, this association is non-significant once chronic NSAID use is taken into account. Use of COX-2 inhibitors has the highest association with development of IHD in patients with AS. Of note, patients with more severe AS may be more prone to use higher doses of NSAID, which may skew the results of studies that attempt to separate NSAID use from development of IHD [37,39,40].

HLA-B27 correlation with CV morbidity

HLA-B27 is associated with AS but the presence of one or two positive HLA-B27 alleles is not associated with the development of cardiac conduction abnormalities. Also, according to a recent cross-sectional study, HLA-B27 does not have any impact on CV events in AS [41].

AS and all-cause mortality

Lehtinen et al. observed that overall mortality in patients with AS is approximately 1.6 to 1.9 times higher than the general population. Upon further inspection of the causes of death in 152 out of the 398 patients with AS studied for an average duration of 25.7 years, 27 patient deaths were related to AS. More specifically, 19 patients died from secondary amyloidosis, six from CV complications, and one patient from spinal fracture [42]. Another study involving 677 patients with AS calculated SMRs. Circulatory disease was the most frequent cause of death (40.0%), followed by malignancy (26.8%), and infections (23.2%). Factors independently associated with reduced survival in patients with AS were diagnostic delay (OR 1.05), increasing levels of CRP (OR 2.68), work disability (OR 3.65), and not using any NSAIDS (OR 4.35) [42,43].

AS and hypertension

Hypertension (HTN) is more common in AS than in an age-adjusted and comorbidity-adjusted comparison group (23.2% vs. 17.8%). Around 70% of patients with AS also have HTN. Longstanding HTN is an independent risk factor for CV disease [1].

AS, atrioventricular block, and aortic insufficiency

AS is associated with elevated rates of cardiac conduction abnormalities, which contribute to the increased mortality rate in patients with AS. Abnormalities in cardiac conduction are not markers of the extent of disease activity or patients’ functional status in patients with AS or limited to particular HLA-B27 subtypes. The HLA-B27 subtype B270502 is common in patients with AS, but the presence of a single allele or two alleles has no relationship with the existence of cardiac conduction abnormalities. The etiology of cardiac conduction abnormalities in AS is obliterative (occlusive) endarteritis of the small blood vessels that supply the atrio-ventricular (AV) node and the aortic root, which leads to the development of AV block, arrhythmias as well as aortic valve insufficiency and resultant aortic regurgitation murmur. Obliterative endarteritis of the small blood vessels that supply the affected joints in AS is the same etiology of development of AV block and aortic valve insufficiency. In patients with AS, even without clinical evidence of heart disease, the aortic roots are less elastic than in healthy controls, based upon measurement of aortic distensibility. AV blocks (second degree Mobitz Type II and third degree) require pacemaker placement for treatment, and aortic valve insufficiency may require repair or replacement if severe. The proportion of patients with AS who have coronary artery disease and hypertension are similar in patients both with and without cardiac conduction abnormalities [44].

Some studies show that cardiac conduction anomalies in AS may be associated with male sex, higher disease activity in addition to CV disease risk factors [45]. Increased prevalence of bradycardia and longer QTc intervals in patients with AS has also been reported [46].

As per the study done by Goulenok and associates, routine electrocardiogram (ECG) in patients with AS does not show increased incidence of CV abnormality [47]. However, since cardiac conduction abnormalities are common in AS and may present with vague symptoms like shortness of breath, fatigue, and decreased exercise tolerance, all patients with AS who present with vague symptoms should be evaluated with an ECG to assess for cardiac conduction abnormalities as the etiology of their symptoms [44].

AS and atrial fibrillation

AS causes prolonged PQ-interval, which leads to an elevated risk for development of atrial fibrillation and first degree AV-block. Atrial fibrillation doubles the mortality risk, increases the risk for stroke five times, and also increases the risk for heart failure [44,47].

## Conclusions

AS is associated with elevated CV mortality. Changes in blood vessel shape and functionality have been noted to be present in patients with AS and may contribute to the acceleration of atherosclerosis. Patients with AS have an inherent systemic inflammatory process with elevated inflammatory markers that mediate tissue injury and inflammation and potentially encourage the formation of atheromas. Inflammatory markers affect the hypothalamus-pituitary-adrenal (HPA) axis, which influences cardiac risk factors including insulin sensitivity, elevated BMI, and elevated blood pressure. In light of the link between inflammation in AS and worsening CV mortality, the use of anti-inflammatory agents is a subject of considerable interest for both treating AS and mitigating the development of CV sequelae. Chronic NSAID use is a mainstay for the treatment of AS and is associated with an increased risk of development of IHD. The underlying cause of this elevated cardiac mortality has been difficult to identify as patients with AS are on chronic NSAIDs, which are independently associated with elevated cardiac mortality risk. AS is also associated with increased cardiac conduction abnormalities, which also contribute to increased mortality. Symptomatic AS patients should undergo ECG and other relevant investigations for detection of cardiac conduction abnormalities; however, routine ECG in asymptomatic patients is not helpful in this regard.
